# Beware the Anomalous Portal Vein

**DOI:** 10.1155/1994/16017

**Published:** 1994

**Authors:** M. K. Koh, H. Ahmad, P. Watanapa, R. P. Jalleh, N. A. Habib

**Affiliations:** Department of Surgery Royal Postgraduate Medical Hammersmith Hospital Du Cane Road London W12 0NN UK

## Abstract

Portal vein thrombosis is an unusual potential complication of liver resection. In our case it was due to ligation of the right branch of the portal vein during right hepatectomy in a patient without portal vein bifurcation. Hepatic angiography can delineate this abnormality and influence the choice of surgical management.


*HPB Surgery*, 1994, Vol. 7, pp. 237–240
Reprints available directly from the publisher
Photocopying permitted by license only

© 1994 Harwood Academic Publishers GmbH
Printed in the United States of America

### CASE REPORT

### BEWARE THE ANOMALOUS PORTAL VEIN

M.K. KOH, H. AHMAD, P. WATANAPA, R.P. JALLEH and N.A. HABIB
*Department of Surgery, Royal Postgraduate Medical, Hammersmith Hospital, Du Cane Road, London W12 0NN, UK*

*(Received 11 November 1992)*

Portal vein thrombosis is an unusual potential complication of liver resection. In our case it was due to ligation of the right branch of the portal vein during right hepatectomy in a patient without portal vein bifurcation. Hepatic angiography can delineate this abnormality and influence the choice of surgical management.

KEY WORDS: Portal vein anomaly, liver resection, portal vein thrombosis

### CASE REPORT

A 45-year old woman was referred to Hammersmith Hospital with a three month history of obstructive jaundice and recurrent cholangitis due to a cystic lesion in the liver. Ultrasound and computerised tomography revealed a cystic mass (in segments IV, V and VII) with intrahepatic biliary dilatation. The radiological features were consistent with Caroli's disease of the liver. ERCP and PTC confirmed communication of the cyst with the biliary tract. A percutaneous drainage followed by internal-external biliary stenting and antibiotic therapy failed to resolve the cholangitic attacks and obliterate the abscess cavity. Liver resection was then thought to be the most appropriate treatment to remove the source of unremitting chronic infection. At laparotomy, resection of the cyst was performed with a right hepatectomy extended to the quadrate lobe. There were multiple calculi within the cyst. Postoperatively, the patient was stable in the intensive care unit for 36 hours, after which she developed septicaemia and disseminated intravascular coagulation (DIC). Biochemical parameters were: WBC 7.1 $\times$ $10^9$/L, and platelets 35 $\times$ $10^9$/L; PT 28 secs., APTT 46 secs., TT 15 secs; fibrinogen degradation product $<$ 256 mg/L and fibrinogen 1.3g/L; Liver function tests — AST 333 iu/L, bilirubin 162 mmol/L and albumin 44g/L. She then developed a sustained drop in systemic vascular resistance and blood pressure and slipped into coma. An urgent CT scan excluded intracranial bleeding or cerebral oedema. On the tenth post-operative day visceral angiography revealed complete thrombosis of the portal vein. She continued to deteriorate despite intensive ionotropic support and died shortly.

Address correspondence to: N.A. Habib, Department of Surgery, Royal Postgraduate Medical School, London W12 0NN, United Kingdom

Postmortem examination confirmed portal vein thrombosis and widespread necrotic liver parenchyma.

### CONCLUSION

Portal vein thrombosis following partial hepatectomy is a rare occurrence. It is the first time this complication has occurred in a personal series of 120 consecutive hepatectomies (NAH). The diagnosis of portal vein thrombosis was missed in this patient during the early post-operative period. Septicaemia was considered the primary diagnosis with massive resection in a chronically infected liver thought to be the contributing factor. A combination of liver failure, hepatic coma, DIC and decreased systemic vascular resistance should alert the clinician to the possibility of portal vein thrombosis. Clamping of the main portal vein during liver resection has not been reported to cause portal vein thrombosis. Close scrutiny of pre and post-operative angiograms (Figure 1) in this patient showed congenital absence of the main left branch of the portal vein. Portal venous supply to the left liver was through retrograde filling from the right lobe. This vascular anomaly occurs in one percent of the population as suggested by Couinaud's study of 103 postmortem examinations[2]. Agosson-Voyeme encountered one such case in a series of 62 cases[1]. If portal vein thrombosis was diagnosed earlier, this patient would have survived

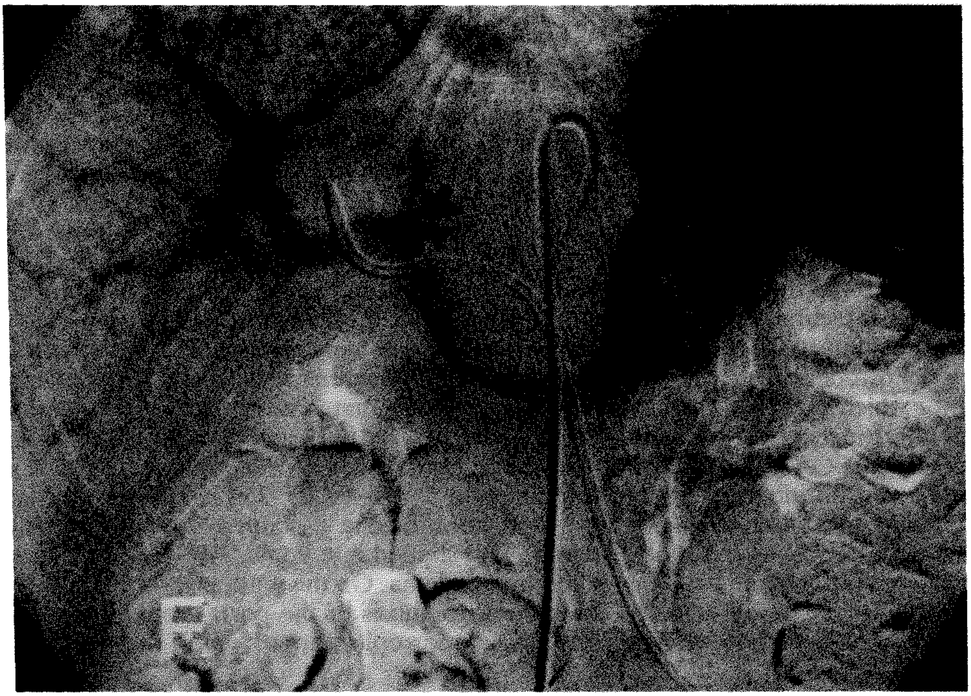

**Figure 1** Preoperative indirect venous splenoportogram showed a patent portal vein with absence of left main portal branch (arrowed). This was replaced by two small branches.

with urgent liver transplantation[3]. The late diagnosis excluded this option as by that time the patient was severely septic, with multiple organ failure. Portal vein thrombectomy was not appropriate here since there was no sizeable left portal vein. Revascularization of the liver via the umbilical vein was theoretically possible but would have been extremely difficult to perform in a rather ill patient. This case exemplifies a rare but important anatomical anomaly of the portal vein that all surgeons should be aware of.

### *References*

1. A.K. Agosson-Voyeme (1982) La segmentation hepatique en romodensitometrie. Paris These 3e Cycle 1982.
2. C. Couinaud (1953) Etude Sur la veine intrahepatique. *Presse Med.*, 61e annee no 70 pp. 1434–1438
3. Stieber, A.C. Zetti, G., Todo, S., Tzakis, A.G., Fung, J.J., Marino, I., Casavilla, A, Selby, R.R. and Starzl, T.E. (1991) The spectrum of portal vein thrombosis in liver transplantation. *Ann. Surg.*, **213**, 199–206

*(Accepted by S. Bengmark 7 March 1993)*

### INVITED COMMENTARY

I reported the first case of absence of the portal bifurcation in 1957[1]. I more closely studied this anomaly in my book issued in 1989[2], and reported Agossou's case. Since then I found another case published by Hardy[3]. The case of the authors is the first recognized *in vivo*. This makes a total of 4 cases in a total of 330 livers, that is 1,20%.

Though quite uncommon, this eventuality should always be researched, as the consequences, if a right hepatectomy is contemplated, are extremely serious. It makes also bipartition for transplantation impossible, as well as procurment of a transplant from a living donor. The portal bifurcation should always be investigated by portography or ultra-sonography.

C. Couinaud
Paris

### *References*

1. C. Couinaud (1957) Le foie. Etudes anatomiques et chirurgicales, Paris, Masson
2. C. Couinaud (1989) Surgical anatomy of the liver revisited, Paris pers. ed.
3. K.J. Hardy and D.H. Nye (1969) An abnormal portal vein. *Surgery*, **66**, 676–678

### INVITED COMMENTARY

Portal vein thrombosis is a rare event in liver resection. It is usually the result of technical mishaps, such as inadvertent ligation of one of the main portal vein branches to the remaining hepatic stump. There is no question that portal vein malformations would theoretically predispose to technical mishaps, but these should be easily avoided with prior knowledge of the presence of the malformation. Although not all groups routinely perform traditional angiographic studies prior to tumor resection, most do. The ones that do not, normally perform a CT portogram or MRI study that would easily allow identification of any major anomaly.

Rapid deterioration of the patient's status with the picture of acute liver failure should immediately alert the surgeon to the possibility of a vascular catastrophe. A Doppler ultrasound study of the liver should be immediately performed, since it is a non invasive and easily performed study. Once an early diagnosis of either portal or portal vein or hepatic artery thrombosis has been established, the patient should be immediately considered for emergent liver transplantation, since otherwise the survival is almost always nil. Retrospectively, this particular patient would have probably benefited from a liver transplant from the very beginning since a resection was not technically feasible.

A. Stiber
Atlanta

